# Systems metabolic engineering for citric acid production by *Aspergillus niger* in the post-genomic era

**DOI:** 10.1186/s12934-019-1064-6

**Published:** 2019-02-04

**Authors:** Zhenyu Tong, Xiaomei Zheng, Yi Tong, Yong-Cheng Shi, Jibin Sun

**Affiliations:** 1 Department of Grain Science and Industry, Kansas State University, Manhattan, KS 66506 USA; 2 Tianjin Institute of Industrial Biotechnology, Chinese Academy of Sciences, Tianjin, 300308 People’s Republic of China; 3 Key Laboratory of Systems Microbial Biotechnology, Chinese Academy of Sciences, Tianjin, 300308 People’s Republic of China; 4 COFCO Biochemical (Anhui) Co. Ltd, Bengbu, 233000 People’s Republic of China

**Keywords:** *Aspergillus niger*, Citric acid, Systems biology, Metabolic engineering, CRISPR/Cas9 genome editing

## Abstract

Citric acid is the world’s largest consumed organic acid and is widely used in beverage, food and pharmaceutical industries. *Aspergillus niger* is the main industrial workhorse for citric acid production. Since the release of the genome sequence, extensive multi-omic data are being rapidly obtained, which greatly boost our understanding of the citric acid accumulation mechanism in *A. niger* to a molecular and system level. Most recently, the rapid development of CRISPR/Cas9 system facilitates highly efficient genome-scale genetic perturbation in *A. niger*. In this review, we summarize the impact of systems biology on the citric acid molecular regulatory mechanisms, the advances in metabolic engineering strategies for enhancing citric acid production and discuss the development and application of CRISPR/Cas9 systems for genome editing in *A. niger*. We believe that future systems metabolic engineering efforts will redesign and engineer *A. niger* as a highly optimized cell factory for industrial citric acid production.

**Electronic supplementary material:**

The online version of this article (10.1186/s12934-019-1064-6) contains supplementary material, which is available to authorized users.

## Background

Citric acid (2-hydroxy-propane-1,2,3-tricarboxylic acid) is known as an intermediate of the tricarboxylic acid cycle that is used to release energy from carbohydrates, fats, and proteins via the oxidation of acetyl-CoA [1, 2]. Citric acid is also the most important bulk product in the organic acid industry, owing to its ubiquitous applications, including beverage and food, pharmaceutical, detergents, cosmetics and organic chemical industries [1, 2]. Citric acid is widely used as an ingredient in carbonated drinks, an acidulant, and flavor additive, due to its pleasant taste, palatability and low toxicity. Moreover, citric acid is also used as a chelating agent and detergent for metal finishing and cleaning, lubricants, animal feeds and plasticizers. The various industrial application promotes the worldwide market of citric acid reached up to 1.7 million tons in 2007, with an annual increase of 3.5–4.0% [3].

The high commercial interest has attracted numerous scientists to devote themselves to citric acid over-producing strain development since the last century. Many microorganisms have been discovered to accumulate citric acid including *Absidia* sp., *Acremonium*, *Botrytis*, *Eupenicillium*, *Penicillium*, and some *Aspergillus* sp., such as *Aspergillus niger*, *Aspergillus awamori*, *Aspergillus nidulans*, *Aspergillus luchensis*, and *Aspergillus flavus* [4]. Besides filamentous fungi, some bacteria and yeast strains are also found to produce citric acid. Bacteria have been reported, including *Bacillus* sp., *Brevibacterium* sp., *Corynebacterium* sp., *Klebsiella* sp., and *Pseudomonas* sp. amongst others [3]. Yeast strains are found to be potential producers from a variety of carbon sources, such as *Candida* sp. and *Yarrowia* sp. [4]. However, due to large amounts of by-product iso-citric acid during yeast fermentation [4], approximately 80% of world-wide citric acid is produced by submerged fermentation using *A. niger* [5].

Although many microorganisms could be utilized for citric acid production, since 1917, Currie discovered that some *A. niger* strains excreted large amounts of citric acid at the initial pH of 2.5 [4]. *A. niger* has been the main industrial workhorse owing to its unique inherent physiological characters and better fitness for industrial fermentation [2, 4, 6]. *A. niger* has powerful polymer degrading enzyme systems to hydrolyze many polymeric substrates, enabling to rapidly grow and ferment on a variety of low-cost raw materials such as corn meal and molasses [7]. *A. niger* demonstrates great robustness to extreme acid environments, leading to outcompeting other rival microorganisms and reducing contamination risk. The high citric acid yield of 0.95 g/g supplied sugar can be achieved with the assistance of fermentation optimization [2]. However, the theoretical citric acid yield was 1.067 g/g glucose [4], thereby a gap still exists between the practical yield of citric acid and theoretical yield. Under the increasingly fierce competition, demanding for high yield, titer and productivity are crucial for strain development, ultimately, to reduce production costs and minimize environmental problems, as successfully achieved in *Thermotoga maritima* by increasing the H_2_ yield even beyond the previously predicted biological limit [8]. Nevertheless, until recently, the strain development efforts commonly occur through random mutagenesis and screening processes that supplied several mutants with great industrial performance, which constitutes a bottleneck for further improvement, as often the inherent accumulation of detrimental mutations and the precise mutations that lead to strain improvement remain unknown [9]. Obviously, the comprehensive understanding of the complex pathway network with metabolic and transcriptional regulation is prerequisite to achieve strain engineering through a global genome modification [10, 11].

Since 2007, the public release of genome data for *A. niger* strains brought the study of *A. niger* into the post-genomic era [12]. With the rapid development of systems biology and genome editing techniques, the underlying molecular mechanism of *A. niger* citric acid fermentation can gradually be unveiled, and systems metabolic engineering is currently being used to redesign and optimize *A. niger* as a cell factory. Until now, no review has focused on how advances in systems biology and metabolic engineering of *A. niger* are enhancing citric acid production. In this review, we summarize the impact of systems biology on understanding the citric acid molecular regulatory mechanisms, the existing metabolic engineering strategies implemented to improve citric acid production and review the development of CRISPR/Cas9 systems for genome editing in *A. niger*. We also proposed future prospects in the systems metabolic engineering cycle, combining the genome information, modern bioinformatics approaches and efficient molecular genetic manipulation tools, to design and engineer *A. niger* as a highly optimized cell factory for improving the yield, titer and productivity with reduced costs and improved environmental sustainability.

### Systems biology boosts the understanding of citric acid metabolic regulation in *A. niger*

Citric acid is the first intermediate of the TCA cycle, and is synthesized by the condensation of acetyl-coenzyme A (acetyl-CoA) and oxaloacetate moiety [1]. Acetyl-CoA is converted from pyruvate with 1 mol CO_2_ released in the mitochondria, while oxaloacetate is formed by pyruvate carboxylation from pyruvate with 1 mol CO_2_ fixation in the cytoplasm. Oxaloacetate is subsequently converted into malic acid and enters the mitochondria through a malate–citrate shuttle. Malic acid is reconverted into oxaloacetate and oxaloacetate takes part in citric acid synthesis. One mole glucose is converted into 1 mol citric acid with 1 mol of ATP and 3 mol of nicotinamide adenine dinucleotide (NADH), resulting in the maximum theoretical yield of 1.067 g/g glucose [4].

As an intermediate of the TCA cycle, citric acid is commonly catabolized by cis-aconitase, and citrate and ATP usually have feedback inhibition against the glycolysis pathway. Nevertheless, *A. niger* is capable of citric acid accumulation in large amounts by an active glycolytic pathway. The unique citric acid metabolism regulation in *A. niger* has attracted much interest, and several excellent reviews have discussed the biochemical mechanisms before the *A. niger* genome release [1, 2, 4]. Multi-omics data of *A. niger*, including genomics, transcriptomics, proteomics and metabolomics, which are being rapidly obtained and boost our understanding of *A. niger* further to a system and molecular level. The impact of these datasets is discussed in detail in the subsequent section.

### Genomics

The genome contains all the genetic information of an organism, and genome sequencing paves the way for all genes structure and function analyses in addition to the generation of genome-scale metabolic networks. Until now, several genomes of *A. niger* stains with different phenotypes have been submitted in the Genome database of National Center for Biotechnology Information (NCBI, Additional file 1: Table S1). The first genome sequence of an *A. niger* strain, an industrial glucoamylase producer CBS513.88, was published in 2007 [12]. Next followed comparative genomics analyses, for example, the wild type citric acid producer ATCC1015 compared with CBS513.88 [13], and citric acid over-producer H915-1 compared with two degenerated isolates L2 and A1 [14].

Comparative genomics generates new insights to identify the relationship between the genotype and phenotype and uncover the diversity of the strains with specific traits. Specifically, compared to enzyme producer CBS513.88, the genome of the acidogenic wild-type strain ATCC1015 contained about 510 unique genes and a large number of polymorphisms (8 ± 16 SNPs/kb) [13]. The unique genes were 396/510 evenly distributed over the seven chromosomes of CBS513.88 and ATCC1015, respectively. Remarkably, unique genes in CBS513.88 included two alpha-amylases, which were horizontally transferred from *Aspergillus oryzae* to give the amylase over-production phenotype to CBS513.88 [13]. Additionally, the unique genes in ATCC1015 were not directly relevant to citric acid production. There were 3/4 unique putative polyketide synthase encoding genes found in CBS513.88/ATCC1015, which likely explains the different secondary metabolites between the strains [13]. Many mutations with SNPs were found to be relevant for citric acid production, whose function enriched in the plasma membrane-bound ATPase, the γ-aminobutyric acid (GABA) shunt, the TCA cycle, and electron transport chain [10], giving novel insights into potential genome engineering targets.

These comparative genomic studies between different industrial isolates have been further supplemented by genomic profiling of mutant isolates and progenitor strains, which has also been useful for predicting key molecular aspects of citric acid production. For example, comparative genomics of three *A. niger* strains with different citrate production efficiencies and mycelial pellets morphology was analyzed [14]. *A. niger* H915-1 exhibited the highest citrate titer of 157 g/L and the yield of 0.98 g/g total sugar in 85 h with compact pellets and short, swollen hyphal branches, whereas the degenerated isolates A1 and L2 produced 117 g/L in 92 h with less hyphal branch in compact pellets, and 76 g/L in 160 h with mycelial clumps, respectively [14]. Compared to two mutant strains A1 and L2, the most notable mutated genes in hyper-producer H915-1 were found to encode a succinate-semialdehyde dehydrogenase involved in GABA shunt, and an aconitase family protein, which may directly influence the citric acid production [14]. The mycelial pellet morphology is proven to dramatically affect the citric acid fermentation. Interestingly, the hydrophobin and melanin biosynthesis pathway involved in conidial and germ tube aggregation showed no difference among these three strains, while a cell wall protein was absent in H915-1, which might be relevant to the morphogenesis [14]. These discoveries between the mutant isolates provide further possible gene targets for strain improvement, e.g. genes encoding succinate-semialdehyde dehydrogenase, aconitase and cell wall protein.

### Transcriptomics

Transcriptomics is an important technique in functional genomics of *A. niger*. Before the availability of genome data, the first DNA microarray study in *A. niger* only investigated the transcription change of 15 genes [15]. In 2008, Andersen et al. [16] developed a tri-species *Aspergillus* microarray for comparative transcriptomics of *A. niger*, *A. nidulans* and *A. oryzae*. With the availability of microarrays, Salazar et al. [17] elucidated the diversity of glycerol metabolism transcriptional regulation in *Aspergilli* species. Comparative transcriptome has also been applied for unveiling the notable diversity between CBS513.88 and ATCC1015 [13]. Among over 10,000 genes, about 4800 genes showed different transcriptional level between these two strains growing on the same condition. The up-regulated gene cohort in ATCC1015 was enriched in the GO biological functions of electron transport, carbohydrate transport and organic acid transport, ultimately suggesting that these candidate genes could be targeted by over-expression technology to improve citric acid production.

Compared to DNA microarray technologies, RNA sequencing (RNA-seq) is increasingly used for transcriptome analysis, due to its higher sensitivity, accuracy and resolution [18]. After the first transcriptome analysis of the *Aspergillus* genus using RNA-seq was performed in *A. oryzae* [19], Delmas et al. [20] also assessed the genome-wide transcriptional responses to lignocellulose in *A. niger* via RNA-seq. Since then, RNA-seq has been widely used in global gene expression profiling to investigate the transcription response and regulation of *A. niger*, including carbon source utilization and regulation [21–24], conidial and mycelial development [25, 26], cell wall biosynthesis [27, 28], secondary metabolite gene cluster expression [29, 30] and organic acid metabolism [14, 31–33]. Dynamic transcriptomics enables the profiling gene expression across industrially relevant time frames using RNA-seq, shedding light on the transcriptional regulatory mechanisms and ultimately leading to target genes for engineering. Yin et al. [14] obtained the transcriptome data of H915-1 during citric acid fermentation and found that 479 genes showed significant transcription regulation, which involved in the central metabolic pathway, GABA shunt pathway and transporters. With regards to glycolysis in this dataset, only a gene encoding a triose phosphate isomerase was up-regulated, and pyruvate kinase was down-regulated, while most enzymes in the TCA cycle were down-regulated. Compared at the initial stage, ATP-citrate lyase was found to be up-regulated about sevenfold at the citric acid accumulation stage, possibly to generate oxaloacetate from citrate, which then enters the mitochondria and TCA cycle. An additional effect of this use of ATP could be the de-repression of the EMP pathway in an ATP futile cycle, as ATP is known to inhibit enzymes in this pathway, such as the phosphofructokinase (PFK). Taken together, these studies demonstrate how transcriptomic studies have given insight on key traits, citric acid over-production, in the lifestyle and differentiation of *A. niger*.

### Proteomics

Similar to transcriptomics, proteomics is an essential component of systems biology, which enables the qualitative and quantitative assessment of the entire proteins of an organism under different conditions. Lu et al. [34] collected the intra- and extracellular *A. niger* proteome under different carbon substrate using 2-D gel electrophoresis/MALDI-TOF and nano-HPLC MS/MS and found that the secretome was dramatically influenced by the extracellular carbon substrate. Elsewhere, Adav et al. [35] analyzed the *A. niger* protein secretion profile using iTRAQ quantitative proteomics and demonstrated that 102 secreted enzymes ensured the powerful capability and potential of polymer degradation. Moreover, membrane-associated proteomic analysis has been developed to identify the new transporters. Sloothaak analyzed the plasmalemma proteomics under different glucose concentration with a hidden Markov model (HMM) and identified two high-affinity glucose transporter MstG and MstH [36]. They further identified the first eukaryotic l-rhamnose transporter RhtA [37]. These studies provide new strategies to identify the new transporters and improve the transport efficiency of substrate and product.

### Metabolomics and fluxomics

Metabolomics is an important potential tool for industrial biotechnology: uncovering global metabolite profiles, identifying the biosynthetic intermediates and metabolic bottlenecks, elucidating phenotype differentiation, and also previously unknown pathways [38]. A significant body of work has been invested by the metabolomics community for standardizing experimental protocols for maximum reproducibility and non-selective sample preparation methods. These methodological and technical studies, including quantitative assessment of various sampling strategies, quenching approaches, and extraction techniques, are important prerequisites for generating high-quality datasets. Variations in these protocols can dramatically influence the metabolite data quality and their downstream interpretation [39]. Several studies aim to establish reliable and efficient sample preparation methods for *A. niger* metabolomics [40–42].

Early investigations into *A. niger* metabolomics adopted − 45 °C 60% methanol quenching which had previously been applied in yeast [40]. Recently, many groups have demonstrated high methanol concentrations caused lower recovery of intracellular metabolites, and consequently − 20 °C 40% methanol was preferentially used as a quenching solution [42]. However, after comprehensively comparing the impact of fast filtration and cold methanol quenching approaches, we discovered that fast filtration with liquid nitrogen is a further improvement for quenching the cellular metabolism of *A. niger*, given its minimal cell damage, high intracellular metabolite recovery and relatively efficient quenching efficiency [43].

Few intracellular metabolite extraction methods have been used in *A. niger*, such as chloroform/methanol/buffer (CM) [40], or boiling ethanol (BE) [42]. A limitation to these approaches was demonstrated by Jernejc et al. who uncovered that BE displayed lower extraction efficiency of three organic acids (pyruvate, malate and 2-oxoglutarate) compared with the traditional acid and alkaline treatments [41]. Given the extreme acidic and alkalinic extraction methods were not compatible with MS-based detection and global metabolomics analysis, we have recently systemically evaluated seven metabolite extraction methods and unveiled that acetonitrile/water (1:1, v/v) at − 20 °C, combined with boiling ethanol extraction protocols based liquid chromatography-tandem mass spectrometry (LC–MS/MS), showed unbiased metabolite profiling. With aid of this optimal LC–MS/MS metabolomics pipeline, we investigated the dynamics of the metabolite profile over time for a citrate over-producing *A. niger* isolate. The metabolomics analyses suggest that high Embden-Meyerhof pathway (EMP) flux and high level of citric acid precursors ensure citrate accumulation [43]. For example, at the citric acid rapid production stage, the intracellular level of pyruvate and oxaloacetate increased by 5.03- and 12.42-fold, respectively [43].

Similar to the metabolomics, fluxomics analysis is also a powerful strategy to unveil the metabolic properties and in vivo flux distribution in filamentous fungi such as *A. niger*. ^13^C metabolic flux analysis, for example, has been utilized to investigate the metabolic difference in mutant enzyme over-producing strains [44–47]. Pedersen et al. [45] found that the disruption of *oahA* gene encoding oxaloacetate acetylhydrolase in a glucoamylase-producing strain did not influence the central carbon metabolism and metabolic flux distribution, while Driouch et al. [46] discovered that overexpression of fructofuranosidase caused the activation of cytosolic pentose phosphate pathway (PPP) and mitochondrial malic enzyme, suggesting the NADPH supply played an essential role in fructofuranosidase production. Lu et al. [47] also found that the carbon flux to PPP increased in a high glucoamylase-producing strain, compared to the wild type strain CBS513.88. Moreover, combined with the isotope‑assisted metabolomics, they found that the secretion of oxalic acid and citric acid resulted from the higher redox state caused by the imbalance of NADH regeneration and consumption in CBS513.88. Taken together, the integrated analysis of metabolomics and fluxomics will shed light on dynamic changes of metabolites pool and kinetic data of intracellular enzymes, and ultimately, for identifying the limiting metabolic steps.

### Genome-scale metabolic modeling

With the availability of massive multi-omics data [48], genome-scale metabolic modeling plays an important role in integrating multi-omics information and quantitatively analyzing phenotypes, which thereby allows the a priori prediction of an organism’s behavior and the elucidation of molecular mechanisms which underpin these phenotypes [49]. Several metabolic networks of *A. niger* have been developed to uncover the key aspects of citric acid over-production [50–55]. Sun et al. [53] developed the first genome-scale metabolic network of *A. niger* based on the genome information of CBS513.88 and ATCC9029, including enzymes with 988 unique EC numbers, 2443 reactions and 2349 metabolites. Additional copies of alternative mitochondrial oxidoreductase (AOX) and citrate synthase (CS) encoding genes were found in *A. niger*, which might contribute to the citric acid accumulation. Consequently, these open reading frames constitute outstanding candidates for rational strain engineering using the extensive *A. niger* toolkit [56].

Another genome-scale metabolic model *i*MA871 of *A. niger* was reconstructed based on the genome of ATCC1015, including 1190 reactions [51]. Compared to the genome-scale metabolic network described above, this metabolic model is more reliable, including the information of the subcellular localization and transport, which is very important for model simulation. In another study, a dynamic metabolic model was developed by a novel modeling method of dynamic flux balance analysis (dFBA), with the time-course fermentative series of citric acid production, which provided a powerful platform to accurately explore the effects of genetic changes on citric acid fermentation in a dynamic manner [55]. Upton et al. [55] demonstrated that the citric acid accumulation was relevant to the polyphosphate hydrolysis regulation and diauxic growth behavior. The constraint on polyphosphate hydrolysis played a crucial role to initiate the citric acid accumulation by limiting cell growth. These data suggested that the genes involved in polyphosphate and energy metabolism could be new targets to uncover the metabolic change for citric acid accumulation.

In summary, the combination of systems biology datasets from the highlighted studies, key attributes of citric acid accumulation mechanism in *A. niger* can be summarized as follows: efficient carbon utilization and transport resulted from powerful hydrolytic enzyme and glucose transport system, high glycolysis flux resulted from the relief of feedback inhibition of ATP and citrate, high C4 anaplerotic activity catalyzed by pyruvate carboxylase to secure precursor supplement, low cis-aconitase and isocitrate dehydrogenase activity to prevent citrate degradation, efficient alternative respiratory chain mediated by AOX to accelerate NADH oxidation and NAD^+^ regeneration with less energy production, ATP futile cycle and consumption catalyzed by ACL, Mn^2+^ deficiency to keep high glycolysis flux but low citrate degradation flux via TCA cycle, and compact mycelial pellets to secure the oxygen transfer by lower the viscosity of the fermentation broth, and high acid resistance mediate by GABA shunt. With the assistance of systems biology, especially the genome-scale metabolic modeling, it is now possible to identify bottlenecks as targets for *A. niger* metabolic engineering, which efforts to design and optimize new stains capable of enhanced citric acid production on low-cost feedstocks, including agro-industrial wastes and lignocellulose biomass, with reduced energy consumption and environmental contamination.

### Metabolic engineering improves citric acid production in *A. niger*

**Fig. 1:**
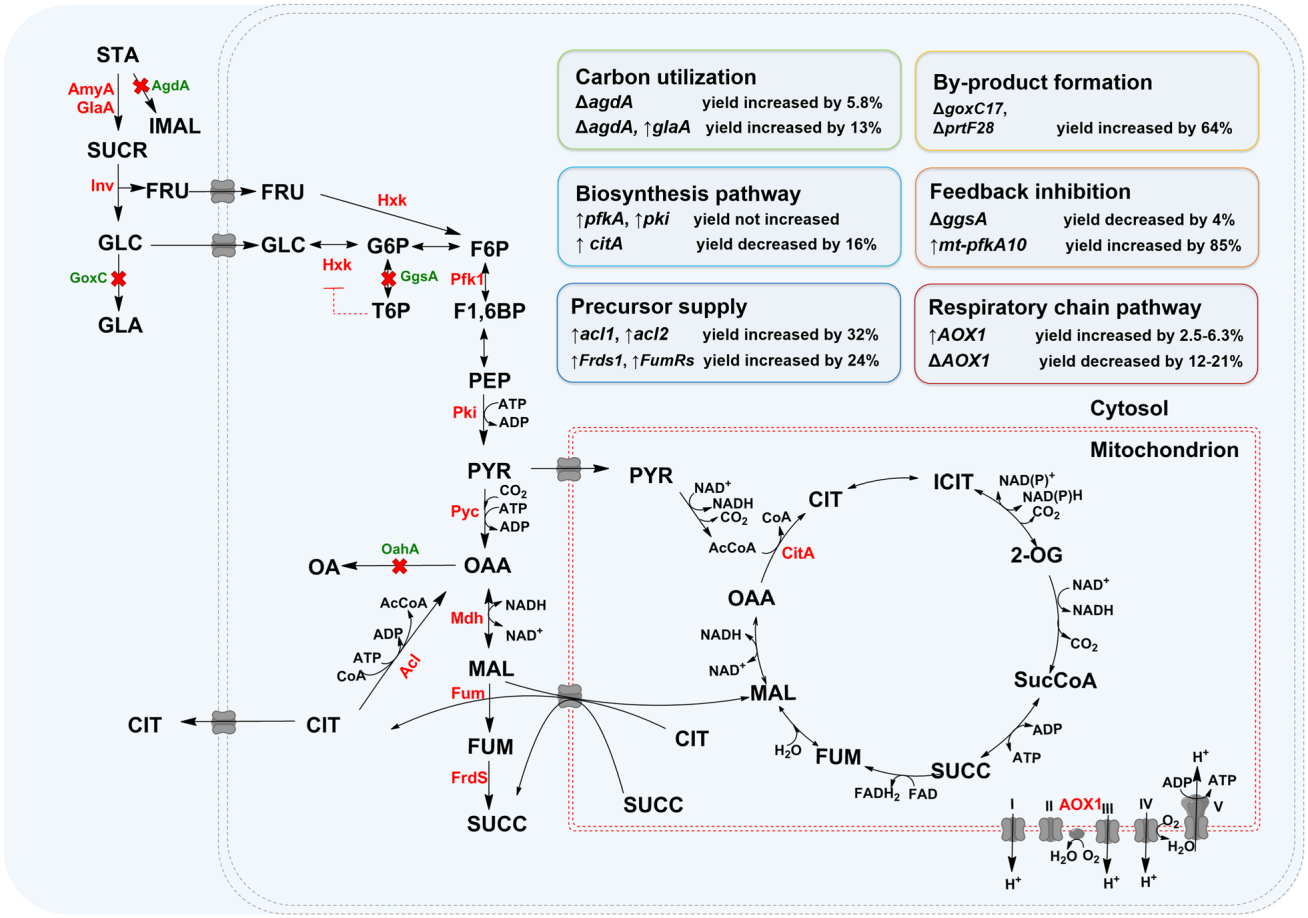
Metabolic engineering strategies for enhancing citric acid production. The central metabolism pathway of *A. niger* was streamlined for citric acid production through engineering of targets for carbon utilization improvement, biosynthesis and precursor enhancement, by-product removal and feedback inhibition reduction and respiratory chain improvement. The Red Crosse represented corresponding gene (green) is deleted. The genes in red represented the targets required to be enhanced. The red dashed line with vertical bar represented the feed-back inhibition, i.e. T6P inhibited the activity of Hxk. *STA* starch, *SUCR* sucrose, *GLC* glucose, *GLA* gluconic acid, *FRU* fructose, *G6P* glucose-6-phosphate, *T6P* trehalose-6-phosphate, *F6P* fructose-6-phosphate, *F-1,6-BP* fructose-1,6-bisphosphate, *PEP* phosphoenol-pyruvate, *PYR* pyruvate, *OAA* oxaloacetate, *AcCOA* acetyl-CoA; *MAL* malate, *OA* oxalic acid, *CIT* citric acid, *ICIT* isocitric acid, *2-OG* 2-oxoglutarate, *SucCOA* succinic CoA, *SUCC* succinate, *FUM* fumarate, *AmyA* amylase, *GlaA* glucoamylase, *Inv* inverase, *AgdA* alpha-1,4-glucosidase, *GoxC* glucose oxidase, *Hxk* hexokinase, *GgsA* trehalose-6-P synthase, *Pfk1* phosphofructokinase, *Pki* pyruvate kinase, *Pyc* pyruvate decarboxylase, *OahA* oxaloacetate acetylhydrolase, *Mdh* malate dehydrogenase, *Fum* fumarase, *Frds* fumarate reductase, *CitA* citrate synthase, *AOX1* alternative mitochondrial oxidoreductase

Along with further deep understanding of citric acid metabolism regulation, instead of traditional mutagenesis, rational metabolic engineering has gradually become a powerful approach to improve citric acid production. The metabolic engineering strategies are summarized in Fig. 1 and Table 1. Compared to the few strategies, e.g. overexpression of invertase [57], inulinase [58], isocitrate lyase [59] and pyruvate carboxylase [60, 61], used in the yeast strain *Y. lipolytica* (Additional file 1: Table S2), the metabolic engineering strategies applied in *A. niger* are more comprehensive, including the enhancement of carbon source utilization, citric acid synthesis, precursor supplements and alternative respiratory chain, the relief of feedback inhibition, the removal of by-products, and so on. Some cases involved in universal strategies, i.e. enhancing the citric acid synthesis [62, 63] and eliminating by-product formation [64], have been reported in the previous review [65]. Herein, we summarized the current metabolic engineering strategies for citric acid production.

**Table 1:** Metabolic engineering strategies for enhancing citric acid production in *A. niger*

Strain	Engineering strategy	Original strain	Titer (g/L)	Productivity (g/L/h)	Yield (g/g sugar consumed)	By-product	Fermentation condition	References
Engineering carbon utilization								
TNA 101ΔagdA	Δ*agdA*	CGMCC10142	172.7, ↑8.68%	2.40	0.96^a^	↓52.95% (residual sugar)	Liquefied corn medium, 180 g/L total sugar, 72 h	[66]
OG1	Δ*agdA*, ↑*glaA*	CGMCC10142	185.7, ↑16.87%	2.58	1.03^a^	↓88.24% (residual sugar)	Liquefied corn medium, 180 g/L total sugar, 72 h	[66]
Enhancing citric acid biosynthesis pathway								
50-2-12	↑*pfkA*, ↑*pki*	NW129/NW131	55, no change	0.33	0.64^b^	–	Synthetic medium, 140 g/L glucose, 168 h	[62]
55-14	↑*citA*	NW129/NW131	46, ↓16.36%	0.27	0.52^b^	–	Synthetic medium, 140 g/L glucose, 168 h	[63]
Enhancing precursor supplement pathway								
*acl1*-*acl2*	↑*acl1*, ↑*acl2*	ATCC1015	42, ↑32%	0.35	0.42^a^	–	Synthetic medium, 100 g/L glucose, 120 h	[67]
Δacl	Δ*acl1*	AB4.1	increased	–	0.043^b^	Succinate, Oxalate, Glycerol	–	[68]
Frds (V)-FumRs	↑*Frds1*, ↑*FumRs*	N402	29.5, ↑59.46%	0.10	0.46^b^	Oxalate	Synthetic medium, 50 g/L glucose, 14 days	[69]
Removal of by-product formation								
NW185	Δ*goxC17*, Δ*prtF28*	NW131	90, ↑63.64%	0.375	0.64^a^	No oxalate	Synthetic medium, 140 g/L glucose, 240 h	[64]
Reducing feedback inhibition								
Δ1–3	Δ*ggsA*	ATCC11414	115, ↓4.17%	–	0.82^a^	–	Shu and Johnson medium, 140 g/L sucrose	[71]
TE23	↑*mt*-*pfkA10*	A158	~120, ↑ ~ 85%	0.4	0.80^a^	–	Synthetic medium, 150 g/L sucrose, ~ 300 h	[72]
Engineering Mn^2+^ response and morphology								
Brsa-25-3	↓*Brsa*-*25*	ATCC11414	2.5, ↑ ~ 35%	0.042	–	–	Synthetic medium, 140 g/L glucose, 60 h in tested tube	[77]
chsC-3	↓*chsC*	CBS513.88	180.3, ↑3.56%	2.50	1.02^a^	–	Liquefied corn medium, 177 g/L total sugar, 72 h	[78]
Regulating the respiratory chain								
CGMCC5751	Adding 0.2 mg/L antimycin A	CGMCC5751	151.67, ↑19.89%	2.11	0.82^a^	–	Liquefied corn medium, 184 g/L total sugar, 72 h	[75]
CGMCC5751	Adding 0.1 mg/L DNP	CGMCC5751	135.78, ↑7.32%	1.89	0.74^a^	–	Liquefied corn medium, 184 g/L total sugar, 72 h	[75]
CGMCC10142-72	↑*AOX1*	CGMCC10142	163.1, ↑2.52%	2.27	0.89^a^	–	Liquefied corn medium, 184 g/L total sugar, with 0.2 μg/mL antimycin A, 72 h	[76]
CGMCC10142-102	↑*AOX1*	CGMCC10142	169.1, ↑6.29%	2.35	0.92^a^	–	Liquefied corn medium, 184 g/L total sugar, with 0.2 μg/mL antimycin A, 72 h	[76]
CGMCC10142-3-4	Δ*AOX1*	CGMCC10142	140.1, ↓11.95%	1.95	0.76^a^	–	Liquefied corn medium, 184 g/L total sugar, with 0.2 μg/mL antimycin A, 72 h	[76]
CGMCC10142-4-10	Δ*AOX1*	CGMCC10142	125.6, ↓20.75%	1.74	0.68^a^	–	Liquefied corn medium, 184 g/L total sugar, with 0.2 μg/mL antimycin A, 72 h	[76]

^a^Yield g/g sugar supplied

^b^Yield g/g sugar reported in the literatures

### Engineering carbon utilization

*Aspergillus niger* is able to secrete a cocktail of hydrolytic enzymes to rapidly degrade complex polymers found in cheap substrates (such as feedstock) into mono-saccharides. However, when liquefied corn starch was utilized for citric acid production, about 2%–3% of the residual sugar remains at the end of the fermentation process. Owing to citric acid production scale of about 1.7 million tons, the residual sugar would account for the annual loss of 150 thousand tons of corn and lead to great environmental pressure world-wide [66]. Therefore, the reduction of residual sugar plays an important role in improving the efficiency of citric acid production. Iso-maltose, synthesized by α-glucosidase, is the main component of the residual sugar in citric acid fermentation broth [66]. Deletion of α-glucosidases encoding gene *agdA* efficiently reduced the isomaltose concentration [66]. Combined with the over-expression of glucoamylase *glaA*, the residual sugar reduced about 88.2% and the citric acid production increased 16.9%, reaching up to 185.7 g/L [66]. The multi-copies of the *glaA* gene under the native P*agdA* promoter improved the extracellular glucoamylase activity by 34.5% [66]. The glucoamylase activity did not strictly positive correlate with citric acid yield, but it significantly influences the saccharification when using corn starch as the raw carbon source [66]. Thus, the increase of glucoamylase activity resulted in higher citric acid production, and constitutes a promising avenue for further biotechnological research.

### Enhancing precursor supplement pathway

Acetyl-CoA and oxaloacetate are the two direct substrates for citric acid synthesis. Acetyl-CoA is generated by pyruvate dehydrogenase (PDH), cytosolic acetyl-CoA synthetase (ACS) and ATP-citrate lyase (ACL), and fatty acids beta-oxidization [67]. The production of acetyl-CoA by ACL consumes citrate, therefore, ACL should be considered not as precursor provider but product consumer. However, the function of ACL is currently unclear. Meijer et al. [68] showed that the deletion of *acl1* in *A. niger* AB4.1 increased the organic acids including succinic acid and citric acid. Chen et al. [67] found that the deletion of two cytosolic ACL subunits (ACL1 and ACL2) in *A. niger* ATCC1015 resulted in decreasing citric acid production, concomitant with diminished asexual conidiogenesis, conidial germination and cell growth. In contrast, the overexpression exhibited reverse effects, suggesting that ACL is beneficial for citric acid accumulation. It was consistent with the time-series transcriptome analyses of citric acid fermentation, which speculated that the cytosolic ACL may involve in the ATP futile cycle [14].

Oxaloacetate is formed by pyruvate carboxylation in the cytoplasm and subsequently converted into malic acid. After entering the mitochondria through a malate–citrate shuttle, malic acid is reconverted into oxaloacetate and oxaloacetate takes part in citric acid synthesis. Therefore, de Jongh and Nielsen engineered the cytosol reductive TCA (rTCA) cycle by inserting heterogeneous malate dehydrogenase, fumarase and fumarate reductase [69]. It was found that overexpression of cytosolic fumarase FumR and cytosolic fumarate reductase Frds1 improved the citric acid yield and productivity, while overexpression of malate dehydrogenase Mdh2 only accelerated the initial production rate [69]. These results demonstrate the potential for introducing entire new biosynthetic pathways in *A. niger*, and highlight how novel industrial capabilities can be developed using systems metabolic engineering and synthetic biology. Indeed, the citric acid metabolic pathway may be entirely re-routed in the future and even synthesized in the cytoplasm instead of mitochondria.

### Reducing feedback inhibition

Hexokinase is strongly inhibited by trehalose 6-phosphate [70]. However, the disruption of trehalose 6-phosphate synthase (*ggsA*) only slightly led to the earlier initiation of citric acid accumulation and the final citric acid production was even reduced compared with the parent strain or multicopy transformant [71]. Legisa and Mattery speculated that assimilation of trehalose activated by cAMP-PKA signaling pathway at the early growth stage might relieve the inhibition of hexokinase, resulting in the glucose metabolism shift from pentose phosphate (PP) pathway to glycolysis, and concomitantly initiated citric acid accumulation [2].

PFK is another crucial controlling step for glycolysis metabolic flux via the allosteric inhibition or activation. ATP and citric acid are the inhibitors of PFK. Spontaneous post-translational modification plays a vital role in keeping the high activity of *A. niger* PFK1 [2]. In the study of Legisa and Mattey, the native PFK1 (85 kDa) was cleaved to an inactive fragment (49 kDa) which could be reactivated by PKA phosphorylation. The shorter PFK1 fragment is not only resistant to citrate inhibition but also more susceptible to positive effectors, such as AMP, ammonium ions and fructose 2,6-bisphosphate, which suppresses the ATP inhibition. Based on this, Capuder et al. [72] designed an active shorter PFK1 fragment *mt*-*pfkA10* with T89D single site mutation to elude the phosphorylation requirement. *A. niger* TE23, constructed by overexpressing the active shorter PFK1 fragment in *A. niger* A158, exhibited citric acid production of 120 g/L at 300 h, about 70% higher than the control strain [72].

### Regulating the respiratory chain

In the citric acid synthesis pathway, the equivalent quantitative conversion of glucose to citric acid generated 1 mol of ATP and 3 mol of NADH. The NADH oxidization cycle by cytochrome-dependent respiration usually generated excess ATP, which strongly feedback inhibited PFK and impaired the glycolysis flux. Thus, when citric acid begins to accumulate, cytochrome-dependent respiration is replaced by the alternative route, which enables the NADH oxidization without concomitant ATP production [1, 4]. Wallrath et al. [73, 74] found that at the initiation of citric acid accumulation, activities of the cytochrome-dependent respiratory enzymes, especially for Complex I, decrease because of Mn^2+^ deficiency, whereas the AOX activity increases. Recently, some oxidative phosphorylation inhibitors, such as succinate-cytochrome c inhibitor antimycin A or the oxidative phosphorylation uncoupler 2,4-dinitrophenol (DNP) [75]. Hou et al. [76] uncovered the overexpression of *aox1* gene improve citric acid production up to 169.1 g/L in the fermentation medium with antimycin A. Obviously, these studies pave the way for combined engineering of the cytochrome-dependent respiratory chain and alternative respiratory chain by promoter engineering.

### Engineering Mn^2+^ response and morphology

Mn^2+^ deficiency plays a crucial role in citric acid accumulation. Mn^2+^ interferes with *A. niger* metabolism in several ways, for example by preventing citrate reutilization, suppressing macromolecular (protein, DNA, triglyceride and phospholipid) synthesis, enhancing protein degradation and intracellular NH4_+_ concentration, altering the ratio of saturated: unsaturated fatty acid in the plasma membrane, modifying the polysaccharide concentration of the cell wall, and influencing morphology [4]. The *Brsa*-25 gene, which encodes a putative amino acid transporter, is involved in the regulation of morphology formation in response to Mn^2+^. Down-regulation of the *Brsa*-*25* expression by antisense RNA reshaped the mycelial pellets and enhanced the citric acid production by 10% [77]. Similarly, RNA interference of the chitin synthase gene (*chsC*) also caused lower proportion of dispersed mycelia in the mycelial pellets and improved citric acid production about 42.6% [78]. Mn^2+^ response and morphology regulation are highly complex, and involve a large number of genes with different functions. Therefore, an efficient multiplex gene editing technology is in an urgent necessity for testing the synergistic effect and interaction of individual genes in a network.

### New generation techniques speed up systems metabolic engineering in *A. niger*

Genetic and genomic manipulations exert a crucial influence in metabolic engineering of *A. niger* [79]. The rapid development of molecular genetic toolbox allows for and speeds up the realization of knowledge-driven, comparative omics-driven, and model-driven target predictions, thereby increasing the implement speed of systems metabolic engineering cycles. However, as described above, several key genes and metabolic pathways have been modified through the traditional transformation techniques to modulate the citric acid production and productivity. Although the gene targeting efficiency is improved in non-homologous end joining (NHEJ) deficient strains [80, 81], the first step for NHEJ deficient host construction, especially for industrially relevant isolates, and modifying genes in a high throughput manner is still very experimentally challenging and time-consuming.

**Fig. 2:**
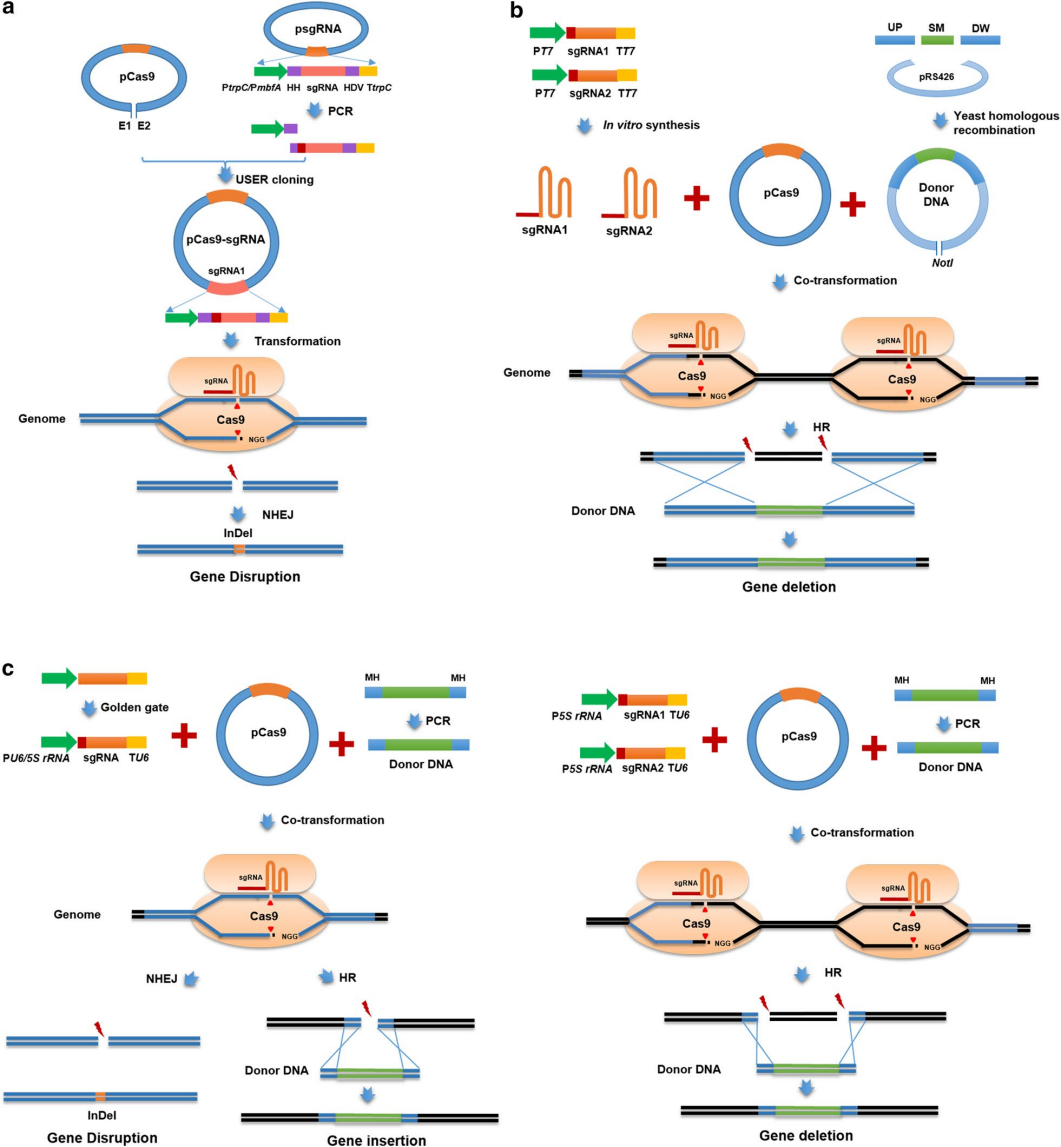
CRISPR/Cas9 genome editing systems used in *A. niger.* **a** CRISPR/Cas9 system based on RNA polymerase II promoters for sgRNA expression enables the NHEJ-mediated gene disruption in *A. niger* [84]. **b** CRISPR/Cas9 system utilizing in vitro transcription for sgRNA synthesis enables the HR-mediated gene deletion with 1.5 kb homologous arm as donor DNA [85, 86]. **c** CRISPR/Cas9 systems based on RNA polymerase III promoters (*U6* and *5S rRNA* promoters) for sgRNA expression facilitate the NHEJ-mediated gene disruption and HR-mediated gene insertion and deletion with 40 bp micro-homologous arms as donor DNA [88, 89]

Clustered Regularly Interspaced Short Palindromic Repeats/CRISPR associated protein (CRISPR/Cas) systems have become a very powerful genome editing technique [82, 83]. Recently, several CRISPR/Cas9 genome editing systems were established in *A. niger* (Fig. 2, Table 2) [84–89]. Nodvig et al. [84] reported the first CRIPSR/Cas9 system in *Aspergilli* sp. (Fig. 2a). They developed all-in-one single plasmid system combined the Cas9 expression cassette with sgRNA expression cassette using RNA polymerase II promoter P*gpdA* in a single vector. To ensure the matured structures of sgRNA, two ribozymes were added the 5′-end and 3′-end of sgRNA. Although the system enables the NHEJ-mediated gene disruption, more cloning efforts and experimental workload are required due to the utilization of ribozymes and sub-cloning of the final single vector. As an alternative solution, Kuivanen et al. [85, 86] adopted in vitro transcription using T7 promoters for sgRNA construction and then co-transformed the sgRNA with the Cas9 expressed plasmid into the protoplasts (Fig. 2b). This system was a suitable approach to achieve instantaneous genome editing, but the efficiency was influenced by the sgRNA stability and uptake [90]. Moreover, this strategy is not suitable for situations where the sgRNA gene needs to be expressed steadily or conditionally, such as CRISPR-AID system mediated transcriptional activation, transcriptional interference, and gene deletion [91]. To fill with the gap of *U6* promoter in *A. niger*, Zheng et al. [88] identified one endogenous *U6* promoter (P*anU6*) and tested the gene disruption efficiency of CRIPSR/Cas9 system based on this P*anU6* and other two heterologous *U6* promoters (P*hU6* and Py*U6*) (Fig. 2c). All the tested U6 promoters enabled guide RNA transcription and the gene disruption, but with low efficiency and few transformants. Zheng et al. [89] developed a novel CRIPSR/Cas9 system using *5S rRNA* gene to promote sgRNA synthesis. Dozens of transformants were obtained and the efficiency was significantly increased with 100% rates of precision gene modifications using short (40-bp) homologous donor DNA (Table 2, Fig. 2c). This system has been applied for chromosome design, as proven by multiplex gene insertion and large DNA fragment deletion to achieve a mycotoxin reduced chassis. This highly efficient CRISPR/Cas9 system facilitates chromosome design in *A. niger*, and allow for the genome manipulations in a high throughput and large-scale manner, thereby increasing the implemented speed of systems metabolic engineering cycle.

**Table 2:** CRISPR/Cas9 genome editing systems used in *A. niger*

sgRNA expression		Gene editing	Donor DNA (SM/Homology arm size, bp)	Efficiency	Advantages	Limitation	References
Promoter	Terminator						
P*gpdA*	T*trpC*	NHEJ-mediated gene disruption	–	Some	Less PAM limitation	Requiring to add HH and HDV, more cloning effort	[84]
In vitro synthesis		HR-mediated gene deletion	*pyrG*/1500	28–100%	Instantaneous genome editing	Hard to be used in gene regulation, dependent on sgRNA uptake and stability	[85]
P*mbfA*	T*trpC*	NHEJ-mediated gene disruption	–	Some	Less PAM limitation	Requiring to add HH and HDV, more cloning effort	[87]
		HR-mediated gene integration	*pyrG/*690 and 834	100%			
P*hU6*	Ploy (T)_6_	NHEJ-mediated gene disruption	–	15%	Less cloning effort	PAM motif (GN_19_GG)	[88]
P*yU6*			–	20%			
P*anU6*			–	23%			
P*anU6*		HR-mediated gene integration	*hph*/40	36%			
5S rRNA	Ploy (T)_6_	NHEJ-mediated gene disruption	–	95.28–100%	High efficiency, less cloning, less PAM limitation	–	[89]
		HR-mediated gene integration and deletion	*hph*/40	100%			
		Multiplex gene editing		45.83%			

### Further prospects

**Fig. 3:**
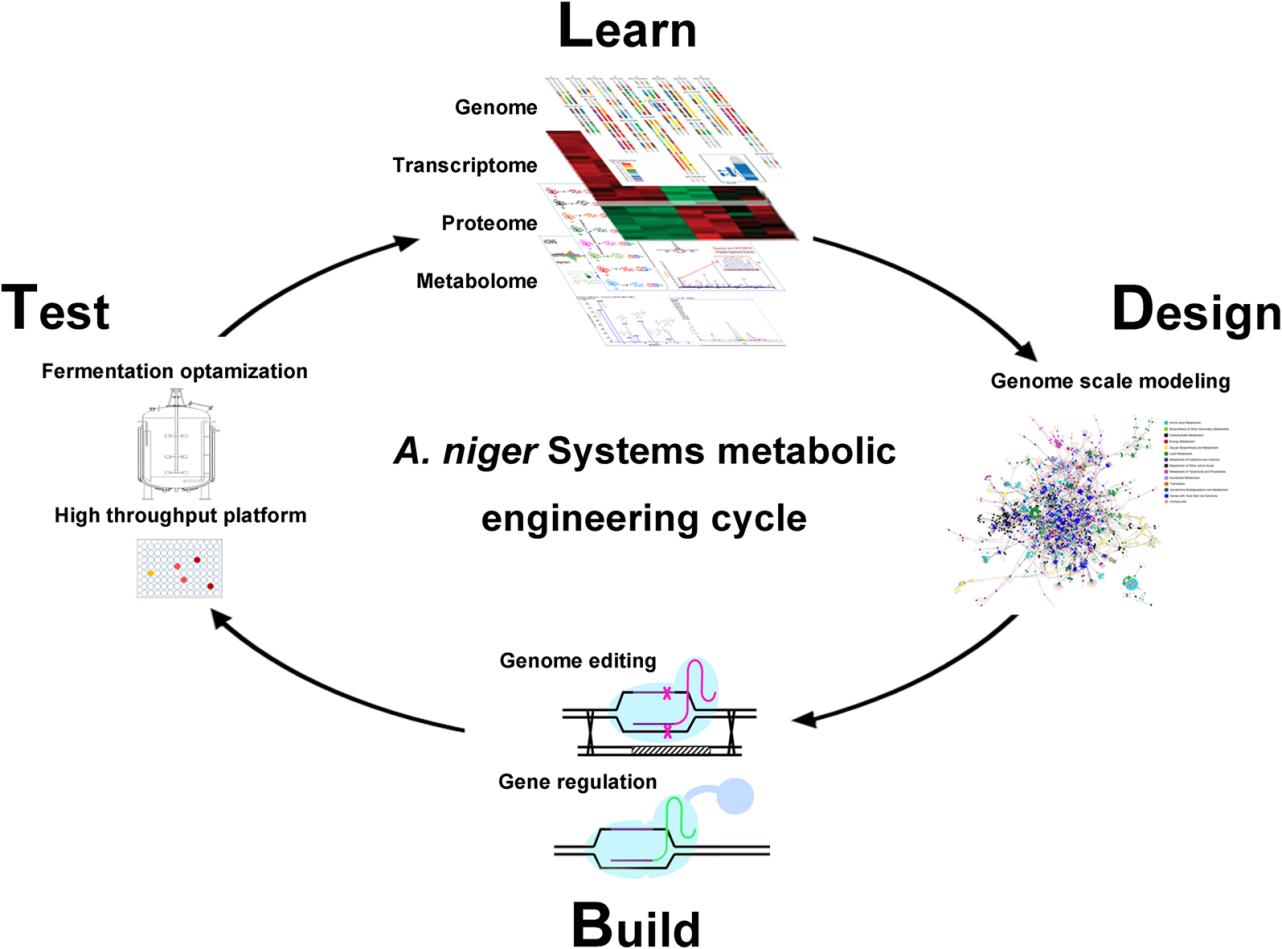
Systemic metabolic engineering of *A. niger* cell factory for citric acid production. A Learn-Design-Build-Test (LDBT) cycle combines multi-omics analysis, computational biology approaches, molecular genetic manipulation toolbox and high through-put platform to achieve customized metabolic engineering on a globe scale of *A. niger.* With the availability of massive multi-omics data of the industrial strains, including genome, transcriptome, proteome and metabolome, genome-scale metabolic modeling could integrate these data (Learn), quantitatively describe the phenotype, and predict the potential targets for metabolic engineering (Design). These targets would be fast verified and combined using the highly efficient genome editing system, and ultimately, obtaining a new generation of cell factories for citric acid production (Build). After detection using high throughput platform and optimization of fermentation processes, the new cell factories have the potential to be industrialized (Test)

As mentioned above, the development of systems biology and genome editing technology paves the way to systemically engineer *A. niger* for citric acid production that is more environmentally friendly, with better food safety, and improved cost-effectiveness. A Learn-Design-Build-Test (LDBT) cycle has been gradually established for customized metabolic engineering on a large-scale of *A. niger*, combining multi-omics analysis, computational biology approaches, molecular genetic manipulation toolbox and high through-put platform (Fig. 3). Namely, all the strategies for protein engineering, pathway engineering and strain engineering result from the deep learning of cell metabolism and regulation based on the genome information and multi-omics data (Learn, Fig. 3). The metabolic engineering targets are designed by knowledge-driven, comparative omics-driven, or in silico model-driven approaches (Design, Fig. 3). The designed *A. niger* strains then would be constructed by genome editing and regulation toolboxes (Build, Fig. 3) and tested by comprehensive detection and fermentation optimization using high throughput platforms (Test, Fig. 3). To implement systems metabolic engineering cycle, we proposed several further challenges and prospects for each stage.

First, the massive multi-omics data provide the feasibility for understanding the *A. niger* at a systems-level. On one hand, to construct the stoichiometric and/or dynamic biological network, more absolutely quantitative omics data are required. On the other hand, the more efficient integrated approaches for multi-omics data are required to uncover the interactions among multi-omics data and the molecular regulation mechanism at different molecular levels, and ultimately to achieve the holistic design of new citric acid producing isolates meeting various requirements.

Secondly, until now, three metabolic engineering strategies have been developed for target prediction, including current knowledge-driven design, comparative omics-driven design, and in silico modeling design. Common knowledge-driven design strategies mostly focus on the improvement of precursor supplements, reduction of by-product formation and feedback inhibition, which are usually limited by the complexity of metabolic regulation. The comparative omics-driven design approach is suitable to distinguish the key genes contributed to specific phenotypes, in which the selection of intercomparable strains and condition design is of vital importance for target discovery. Otherwise, it is hard to find the key genes from the numerous potential differences. In contrast, genome-scale models facilitate to integrate multi-omics data and construct the organism-specific metabolic maps, interpret the changes of transcriptional and metabolic profiles, ultimately to establish a full understanding of the cell regulation complexity at different levels [92]. Most genome-scale metabolic models of *A. niger* have been developed for steady state, usually as stoichiometric models. In the future, a dynamic/kinetic model is required to illustrate the multi-omics and process data and predict the behavior of *A. niger* responding to interior and exterior changes during citric acid fermentation. Therefore, genome-scale metabolic modeling would become the main systems approach to optimize the metabolic engineering design.

Thirdly, to construct on-demand well-designed strains, three aspects should be paid more attention, including construction of synthetic biological module, construction of robust chassis and development of multiplex genetic manipulation toolboxes. CRISPR/Cas9 genome editing technology facilitates the rapid verification of new hypotheses and the realization of target predictions. Multiplex genome engineering and marker-free base editing are required to be established in *A. niger* to accelerate the systems metabolic engineering cycle for final industrialization.

Finally, high throughput platforms, including the spore collection, strain cultivation, metabolite detection and fermentation optimization, should be developed to test and screen the well-designed strains in a large-scale. All the exhaustive measurement data would be applied for the next strategy design.

## Conclusions

With the rapid development of systems biology and synthetic biology, a major goal for the future of *A. niger* biotechnology is the generation of designer strains and super cell factory with higher titre, yield and productivity. Toward this end, some directions for systems metabolic engineering can be summarized as follows: improving the substrate utilization, removing by-product synthesis, removing negative feedback effect, enhancing the precursor supplement, enhancing the transport efficiency of substrates and citric acid, optimizing the NADH regeneration by regulating the respiratory chain, enhancing the robustness and resistance against environmental stress, regulating the morphology to fit the process operation. Many genome editing strategies could be applied to achieve metabolic engineering, including promoter engineering of target genes with inducible promoters, transcription factor engineering, transporter engineering, and transcription regulation via CRSIPRi/CRSIPRa system or RNAi. In conclusion, holistic design from multi-omics analyses and dynamic modeling, genome editing combined with synthetic biology provide great promise for achieving rational design of *A. niger* at a system level.

## Additional file

**Additional file 1: Table S1.** Genome information of several *A. niger* strains. **Table S2.** Metabolic engineering strategies for enhancing citric acid production in *Yarrowia lipolytica.*

## Abbreviations

CRISPR: clustered regularly interspaced short palindromic repeats
Cas: CRISPR-associated proteins
sgRNA: single guide RNA
NHEJ: non-homologous end joining
HR: homologous recombination
NLS: nuclear localization signal
snRNA: small nuclear RNA
SNPs: single nucleotide polymorphisms
RNA-seq: RNA sequencing
PFK: phosphofructokinase
PDH: pyruvate dehydrogenase
ACS: cytosolic acetyl-CoA synthetase
ACL: ATP-citrate lyase
acetyl-CoA: acetyl-coenzyme A
NADH: nicotinamide adenine dinucleotide
GABA: γ-aminobutyric acid
DNP: 2,4-dinitrophenol
