# Supplementary material for: Systems metabolic engineering for citric acid production by Aspergillus niger in the post-genomic era
Source: Microb Cell Fact. 2019 Feb 4;18:28. doi: 10.1186/s12934-019-1064-6 (PMC6362574; doi:10.1186/s12934-019-1064-6)
Supplement: Supplementary file 1 — Additional file 1: Table S1. Genome information of several A. niger strains. Table S2. Metabolic engineering strategies for enhancing citric acid production in Yarrowia lipolytica. [file 12934_2019_1064_MOESM1_ESM.docx]

**Systems metabolic engineering for citric acid production by *Aspergillus niger* in the post-genomic era**

Zhenyu Tong^1†^ Xiaomei Zheng^2,3†^  Yi Tong^4^ Yong-Cheng Shi^1^ Jibin Sun^2,3*^

^1^Department of Grain Science and Industry, Kansas State University, Manhattan, KS 66506, USA

^2^Tianjin Institute of Industrial Biotechnology, Chinese Academy of Sciences, Tianjin 300308, People’s Republic of China

^3^Key Laboratory of Systems Microbial Biotechnology, Chinese Academy of Sciences, Tianjin 300308, People’s Republic of China

^4^COFCO Biochemical (Anhui) Co., Ltd, Bengbu 233000, People’s Republic of China

^*^Correspondance:

Jibin Sun, Tel.: +86-8486 1949, Fax: +86-8486 1943, E-mail: [sun_jb@tib.cas.cn](mailto:sun_jb@tib.cas.cn);

^†^Zhenyu Tong and Xiaomei Zheng contributed equally to this work.

E-mail addresses:

Zhenyu Tong: zhenyutong@ksu.edu

Xiaomei Zheng: zheng_xm@tib.cas.cn

Yi Tong: tongyi@cofco.com

Yong-Cheng Shi: ycshi@ksu.edu

Jibin Sun: sun_jb@tib.cas.cn

**Table S1 Genomes information of *A. niger* strains**

| **Strain** | **Size (Mb)** | **GC%** | **Scaffolds** | **Genes** | **Phenotype** | **Key findings** | **Reference** |
| --- | --- | --- | --- | --- | --- | --- | --- |
| CBS513.88 | 33.90 | 50.4 | 20 | 14097 | Glucoamlyase producer | Enriching many major facilitator superfamily transporters, fungal zinc binuclear cluster transcription factors. Fumonisin and ochratoxin A synthesis gene clusters were identified. | [[1](#_ENREF_1)] |
| ATCC1015 | 34.85 | 50.3 | 24 | 10947 | Wild type citric acid producer | Mutants enriched in the plasma membrane-bound ATPase, the γ -aminobutyric acid (GABA) shunt, the TCA cycle, and electron transport chain. | [[2](#_ENREF_2)] |
| H915-1 | 35.98 | 49.2 | 30 | 10318 | Citric acid hyper-producer | A succinate-semialdehyde dehydrogenase involved in GABA shunt and an aconitase family protein were identified. | [[3](#_ENREF_3)] |
| L2 | 36.45 | 49.2 | 30 | 10433 | Degenerated citric acid producer |  | [[3](#_ENREF_3)] |
| A1 | 34.64 | 50.1 | 319 | 10123 | Degenerated citric acid producer |  | [[3](#_ENREF_3)] |
| [FGSC A1279](https://www.ncbi.nlm.nih.gov/genome/429?genome_assembly_id=351683) | 35.3967 | 49.80 | 405 | - | Secondary metabolite | The global regulator LaeA dramatically influences the secondary metabolite profile. | [[4](#_ENREF_4)] |
| ATCC 9029 | 33.7 |  | 9510 | ~14000 | Gluconate production | Additional copies of alternative mitochondrial oxidoreductase (AOX) and citrate synthase (CS) | [[5](#_ENREF_5)] |
| SH-2 | 34.60 | 50.3 | 349 | - | Aconidial enzyme producer | Lacking Protein disulfide isomerase PrpA. Mutations enriched in genes of cell wall integrity signaling, beta-1,3-glucan synthesis and chitin synthesis. | [[6](#_ENREF_6)] |
| An76 | 34.88 | 49.4 | 669 | 10373 | \ | A complete xylanolytic enzyme system required for xylan degradation and composed of diverse isozymes was secreted in a sequential order. | [[7](#_ENREF_7)] |

**Table S2** **Metabolic engineering strategies for enhancing citric acid production in *Yarrowia lipolytica***

| **Strain** | **Engineering strategy** | **Original strain** | **Titer (g/L)** | **Productivity(g/L/h)** | **Yield (g/g sugar consumed)** | **By-product** | **Fermentation condition** | **Reference** |
| --- | --- | --- | --- | --- | --- | --- | --- | --- |
| **Engineering carbon utilization and by-product reduction** | | | | | | | | |
| H222-S4 (p67ICL1) T5 | **↑SUC2, ↑ICL1** | H222-S4 | 140.0 | 0.73 | 0.82 | 4 g/L Iso-citrate | Synthetic medium, pH6.8  Total 170 g/L sucrose, 191 h | [[8](#_ENREF_8)] |
| 87 | **↑INU1** | SWJ-1b | 68.9 | 0.22 | 0.74 | 4.1 g/L Iso-citrate; 7.1 g/L total sugar | Synthetic medium, pH6.0  100 g/L inulin, 312 h | [[9](#_ENREF_9)] |
| 30 | **ΔACL1, ↑ICL1, ↑INU1** | SWJ-1b | 84.0 | 0.39 | 0.93 | 1.8 g/L Iso-citrate | Synthetic medium, pH6.0  100 g/L inulin, 214 h | [[10](#_ENREF_10)] |
| **Enhancing precursor supplement pathway** | | | | | | | | |
| PG86 | **↑mgPYC** | SWJ-1b | 101.0 | 0.42 | 0.89 | 5.93 g/L reducing sugar | Synthetic medium, pH6.5  120 g/L total glucose, 240 h | [[11](#_ENREF_11)] |
| Δacl | **↑prPYC1** | SWJ-1b | 111.1 | 0.46 | 0.93 | 1.72 g/L reducing sugar | Synthetic medium, pH6.5  120 g/L total glucose, 240 h | [[12](#_ENREF_12)] |

### References

1. Pel HJ, de Winde JH, Archer DB, Dyer PS, Hofmann G, Schaap PJ, Turner G, de Vries RP, Albang R, Albermann K, et al: Genome sequencing and analysis of the versatile cell factory *Aspergillus niger* CBS 513.88. Nat Biotechnol. 2007;25:221-31.

2. Andersen MR, Salazar MP, Schaap PJ, van de Vondervoort PJI, Culley D, Thykaer J, Frisvad JC, Nielsen KF, Albang R, Albermann K, et al: Comparative genomics of citric-acid-producing *Aspergillus niger* ATCC 1015 versus enzyme-producing CBS 513.88. Genome Research. 2011;21:885-97.

3. Yin X, Shin HD, Li J, Du G, Liu L, Chen J: Comparative genomics and transcriptome analysis of *Aspergillus niger* and metabolic engineering for citrate production. Sci Rep. 2017;7:41040.

4. Wang B, Lv Y, Li X, Lin Y, Deng H, Pan L: Profiling of secondary metabolite gene clusters regulated by LaeA in *Aspergillus niger* FGSC A1279 based on genome sequencing and transcriptome analysis. Res Microbiol. 2018;169:67-77.

5. Sun J, Lu X, Rinas U, Zeng A: Metabolic peculiarities of *Aspergillus niger* disclosed by comparative metabolic genomics. Genome Biology. 2007;8:R182.

6. Yin C, Wang B, He P, Lin Y, Pan L: Genomic analysis of the aconidial and high-performance protein producer, industrially relevant *Aspergillus niger* SH2 strain. Gene. 2014;541:107-14.

7. Gong W, Dai L, Zhang H, Zhang L, Wang L: A highly efficient xylan-utilization system in *Aspergillus niger* An76: a functional-proteomics study. Front Microbiol. 2018;9:430.

8. Forster A, Aurich A, Mauersberger S, Barth G: Citric acid production from sucrose using a recombinant strain of the yeast *Yarrowia lipolytica*. Appl Microbiol Biotechnol. 2007;75:1409-17.

9. Liu XY, Chi Z, Liu GL, Wang F, Madzak C, Chi ZM: Inulin hydrolysis and citric acid production from inulin using the surface-engineered *Yarrowia lipolytica* displaying inulinase. Metab Eng. 2010;12:469-76.

10. Liu XY, Chi Z, Liu GL, Madzak C, Chi ZM: Both decrease in ACL1 gene expression and increase in ICL1 gene expression in marine-derived yeast *Yarrowia lipolytica* expressing INU1 gene enhance citric acid production from inulin. Mar Biotechnol (NY). 2013;15:26-36.

11. Tan MJ, Chen X, Wang YK, Liu GL, Chi ZM: Enhanced citric acid production by a yeast *Yarrowia lipolytica* over-expressing a pyruvate carboxylase gene. Bioprocess Biosyst Eng. 2016;39:1289-96.

12. Fu GY, Lu Y, Chi Z, Liu GL, Zhao SF, Jiang H, Chi ZM: Cloning and characterization of a pyruvate carboxylase gene from *Penicillium rubens* and overexpression of the genein the yeast *Yarrowia lipolytica* for enhanced citric acid production. Mar Biotechnol (NY). 2016;18:1-14.
